# Giant Pelvic Cyst Mimicking a Para-Ovarian Cyst: A Diagnostic Challenge With Successful Surgical Excision and Histopathological Correlation

**DOI:** 10.7759/cureus.117017

**Published:** 2026-09-27

**Authors:** Dipen K Kotwal, Nikunj Barvadiya, Nidhi Gheewala, Anand Kumar, Divyeshkumar N Parmar, Mohith VS

**Affiliations:** 1 General Surgery, All India Institute of Medical Sciences, Rajkot, Rajkot, IND; 2 Surgery, All India Institute of Medical Sciences, Rajkot, Rajkot, IND

**Keywords:** exploratory laparotomy, histopathology, mesenteric cyst, paraovarian cyst, paratubal cyst, pelvic cyst, sigmoid mesentery

## Abstract

Mesenteric and paratubal cysts are uncommon benign cystic lesions of the abdomen and pelvis that may pose a considerable diagnostic challenge because of their nonspecific presentation and overlapping radiological features. Large pelvic cysts may closely resemble ovarian, paraovarian, or mesenteric lesions, making preoperative diagnosis difficult. We report the case of a 30-year-old woman presenting with a progressively enlarging pelvic-abdominal cyst associated with lower abdominal pain, weakness, and weight loss. Sequential ultrasonography, magnetic resonance imaging, and contrast-enhanced computed tomography demonstrated a large benign-appearing cystic lesion but failed to determine its exact origin. Exploratory laparotomy performed jointly by the gynecology and general surgery teams revealed a giant pelvic cyst densely adherent to the sigmoid mesentery, left ovary, and left fimbrial end, strongly suggesting a mesenteric origin intraoperatively. Complete excision with left fimbriectomy was performed while preserving the bowel and ovary. Histopathological examination demonstrated a benign paratubal cyst with an attached normal fallopian tube, highlighting the importance of histopathological correlation in complex pelvic cystic lesions. This case emphasizes the limitations of imaging and operative assessment in determining the true origin of large pelvic cysts and underscores the role of complete surgical excision in both diagnosis and treatment

## Introduction

Mesenteric cysts are rare intra-abdominal lesions arising within the mesentery of the gastrointestinal tract, with an estimated incidence of approximately 1 in 100,000 adults. They may occur anywhere along the mesentery from the duodenum to the rectum, although the small bowel mesentery is the most common site, while involvement of the sigmoid mesocolon is distinctly uncommon. Owing to their rarity and diverse anatomical locations, mesenteric cysts continue to present significant diagnostic and therapeutic challenges for surgeons [1-4].

The clinical presentation of mesenteric cysts is highly variable, ranging from asymptomatic incidental findings to chronic abdominal pain, abdominal distension, palpable abdominal masses, intestinal obstruction, volvulus, hemorrhage, infection, rupture, and, rarely, malignant transformation. Because these manifestations are nonspecific, preoperative diagnosis is often difficult, and the definitive diagnosis is frequently established only during surgical exploration [1,2,5-8].

Ultrasonography and contrast-enhanced computed tomography are the primary imaging modalities for evaluating intra-abdominal cystic lesions. However, despite advances in imaging, accurately determining the site of origin of large cystic masses remains challenging. Mesenteric cysts may mimic ovarian, paraovarian, paratubal, retroperitoneal, lymphatic, enteric duplication, and other cystic lesions, making preoperative differentiation difficult. Consequently, histopathological examination following complete surgical excision remains the gold standard for definitive diagnosis [1,3,4,9-12].

Paraovarian and paratubal cysts are uncommon benign adnexal lesions arising from the mesosalpinx or remnants of the Müllerian or Wolffian ducts. Although most are small and asymptomatic, giant lesions may present with abdominal distension, chronic pain, or palpable abdominal masses. Because of their proximity to the ovary, fallopian tube, and pelvic mesentery, they may closely mimic mesenteric cysts on clinical examination and radiological imaging, posing a considerable diagnostic challenge [13-14].

We report a rare case of a giant benign paratubal cyst that was clinically and radiologically diagnosed as a mesenteric cyst. The true origin of the lesion was identified only during surgical exploration and confirmed by histopathological examination. This case highlights the importance of maintaining a broad differential diagnosis when evaluating large intra-abdominal cystic masses and emphasizes the indispensable role of surgery and histopathology in establishing the final diagnosis and guiding appropriate management [1,3,11,13,14].

## Case presentation

Patient information

A 30-year-old female (G1L1P0A0) presented with intermittent lower abdominal pain radiating to the back for several months. The pain had gradually increased in intensity and was associated with generalized weakness, easy fatigability, and an unintentional weight loss of approximately 3 kg over the preceding 18 months. She had first been informed of a pelvic cyst during antenatal ultrasonography nearly one and a half years earlier but remained largely asymptomatic until recent progression of symptoms.

Past history

The patient had regular menstrual cycles without dysmenorrhea, abnormal uterine bleeding, infertility, or chronic pelvic inflammatory disease. She had no previous abdominal surgery. She was a known hepatitis B surface antigen (HBsAg)-positive patient receiving tenofovir 300 mg once daily under regular gastroenterology follow-up. There was no significant family history of gynecological or gastrointestinal malignancy.

Clinical examination

General examination revealed mild pallor. Vital signs were stable. Per-abdominal examination demonstrated a soft abdomen with a large cystic swelling extending from the pelvis to the level of the umbilicus (approximately 17 × 10 cm). The swelling had a smooth surface, well-defined superior margins, and positive fluctuation. The lower border could not be palpated. The swelling became more prominent in the all-fours position, suggesting an intraperitoneal lesion.

Laboratory investigations

The patient's laboratory investigations are presented in Table 1.

**Table 1:** Laboratory investigations

Investigation	Result	Unit	Reference Range
Hemoglobin	9.9	g/dL	12.0-16.0
Total Leukocyte Count	3.44	×10³/µL	4.0-11.0
Platelet Count	330	×10³/µL	150-450
Serum Creatinine	0.6	mg/dL	0.5-1.1
Blood Urea	26	mg/dL	15-40
Sodium	145	mmol/L	135-145
Potassium	4.8	mmol/L	3.5-5.1
Chloride	110	mmol/L	98-107
Lactate Dehydrogenase (LDH)	197	U/L	140-280
Glycated Hemoglobin (HbA1c)	4.4	%	4.0-5.6
CA-125	18.3	U/mL	<35
CA19-9	18.2	U/mL	<37
Carcinoembryonic Antigen (CEA)	1.18	ng/mL	<5
Alpha-Fetoprotein (AFP)	4.77	ng/mL	<10
Inhibin-B	7.2	pg/mL	Within normal premenopausal reference range
Beta-Human Chorionic Gonadotropin (β-hCG)	<1.2	mIU/mL	<5
Thyroid Profile: Total T4/Total T3/Thyroid-stimulating Hormone (TSH)	15.29/ 2.37/0.87	Ug/dl;ng/ml; mIU/L	5.53-11.0; 0.82-1.58; 0.58-6.88
Prolactin	177.9	mIU/L	-

Radiological evaluation

Ultrasonography demonstrated a well-defined unilocular hypoechoic pelvic cyst without internal septations. MRI pelvis revealed a thin-walled T2 hyperintense lesion measuring approximately 10 × 13 × 16.5 cm extending into the lower abdomen without mural nodules, hemorrhage, calcification, or diffusion restriction (Figure 1). The uterus, urinary bladder, and bowel loops were displaced but not invaded. Contrast-enhanced computed tomography demonstrated a 9.5 × 13.5 × 16.8 cm intraperitoneal cyst displacing adjacent bowel loops while preserving surrounding fat planes (Figure 2). Differential diagnoses included mesenteric cyst, paraovarian cyst, exophytic ovarian cyst, peritoneal inclusion cyst, and enteric duplication cyst.

**Figure 1:**
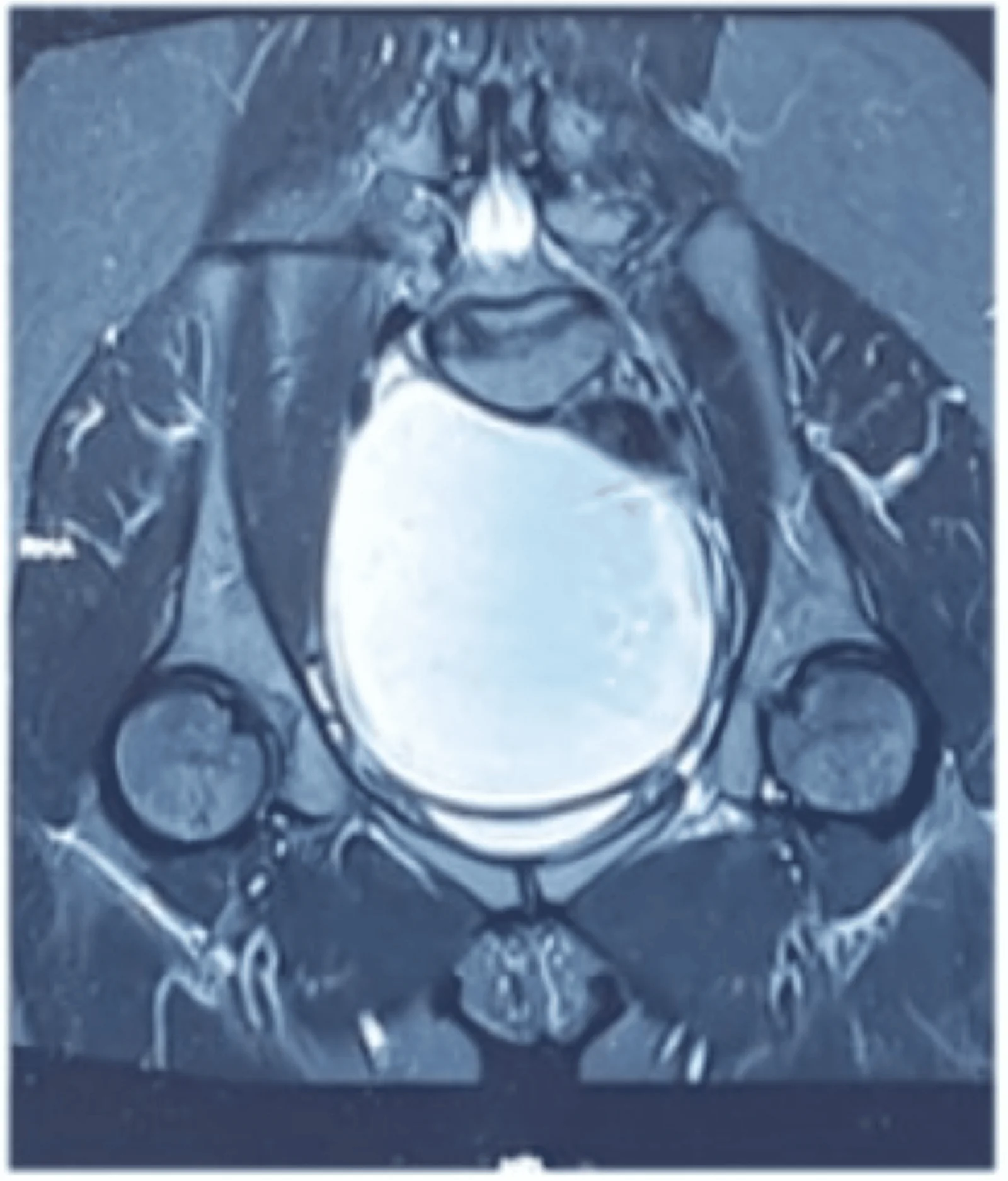
MRI image of the cyst

**Figure 2:**
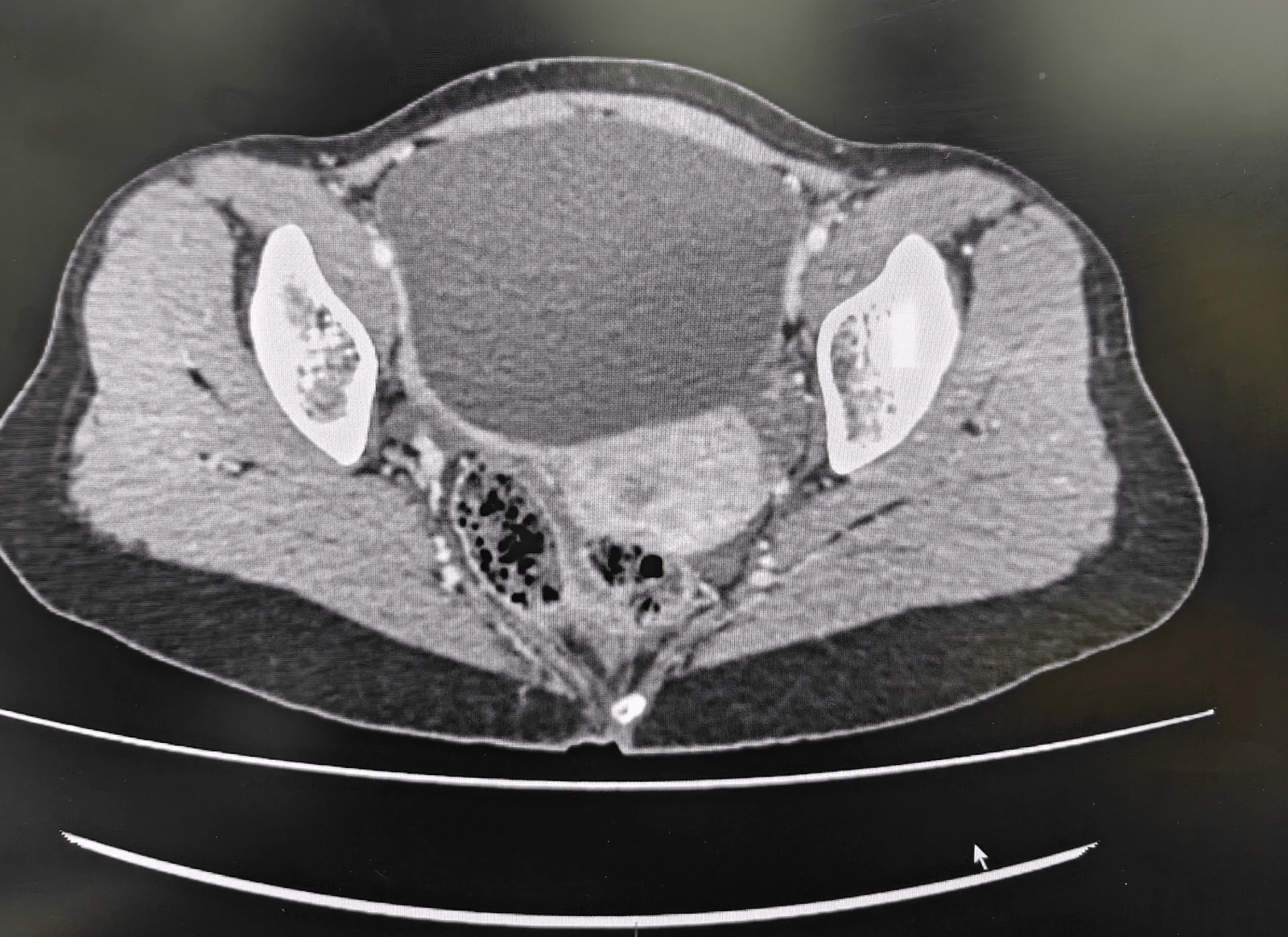
CT image of the cyst

Operative findings

After multidisciplinary discussion between the Departments of General Surgery and Obstetrics & Gynaecology, the patient was taken up for exploratory laparotomy because preoperative imaging could not conclusively establish the origin of the pelvic cyst. A lower midline incision was made, and the peritoneal cavity was entered.

A giant, smooth, tense, thin-walled translucent cyst occupied the pelvis and lower abdomen (Figure 3). The uterus, right ovary, and right fallopian tube were normal. The left fallopian tube was stretched over the surface of the cyst, and its fimbrial end was densely adherent to the cyst wall, giving the appearance of a paraovarian cyst.

**Figure 3:**
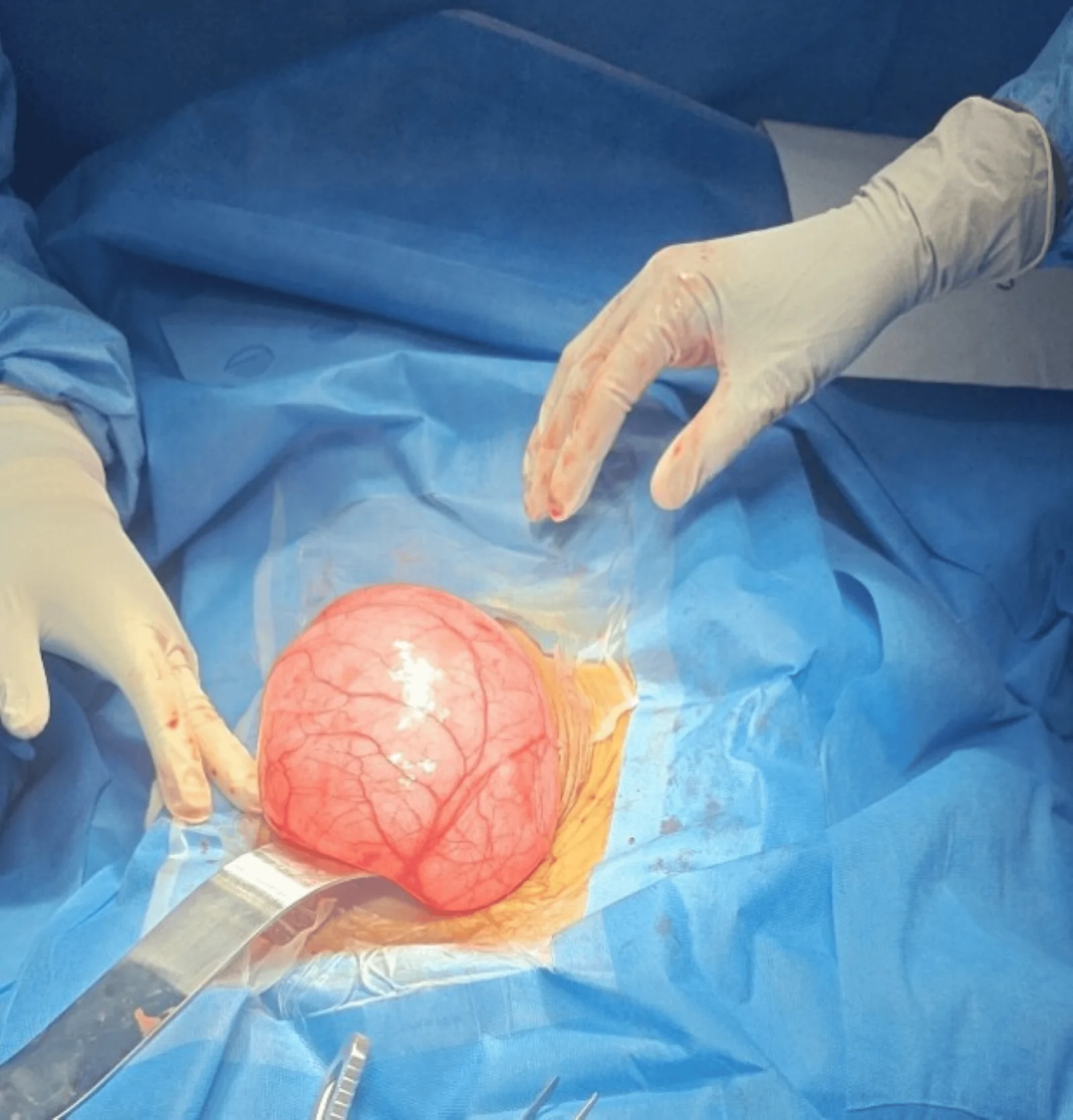
Pelvic cyst after the abdomen was entered

Further exploration by the General Surgery team demonstrated dense adhesions between the cyst, the mesenteric border of the sigmoid colon, the left ovary, and the left fimbrial end (Figure 4). Because of these extensive adhesions, the lesion appeared to originate from the sigmoid mesentery intraoperatively. Careful sharp and blunt adhesiolysis was performed while preserving the sigmoid colon and its vascularity. The left ovary was preserved. The fimbrial end of the left fallopian tube was inseparable from the cyst wall and therefore left fimbriectomy was performed to achieve complete excision.

**Figure 4:**
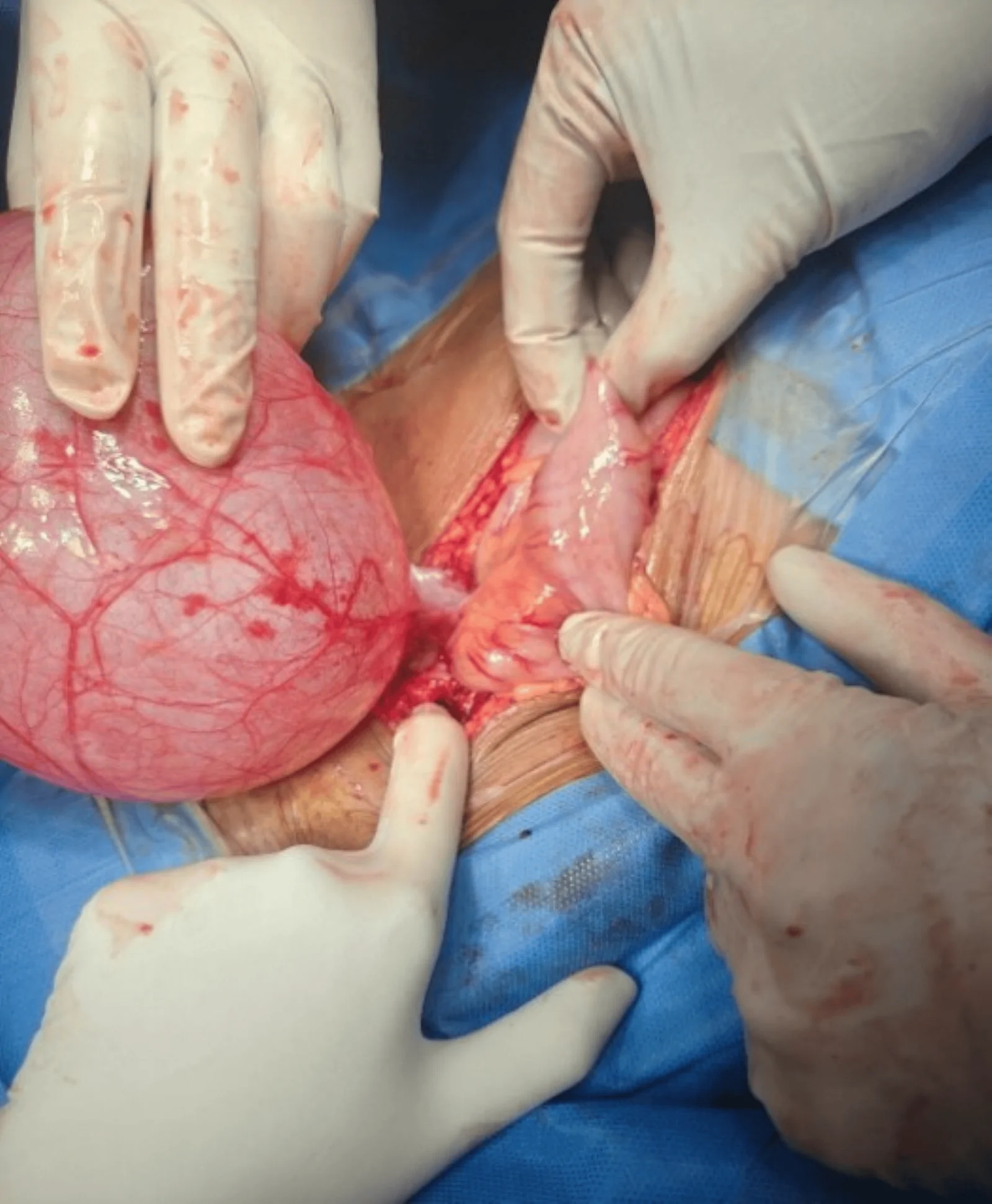
Pelvic cyst attached to the fimbria

The vascular pedicle was individually ligated, and the cyst was excised intact without rupture or spillage. The excised specimen measured approximately 16 × 10 × 6 cm and contained clear serous fluid (Figure 5). The remaining abdominal cavity was systematically inspected, and no additional pathology was identified. Hemostasis was secured, and the abdomen was closed in layers.

**Figure 5:**
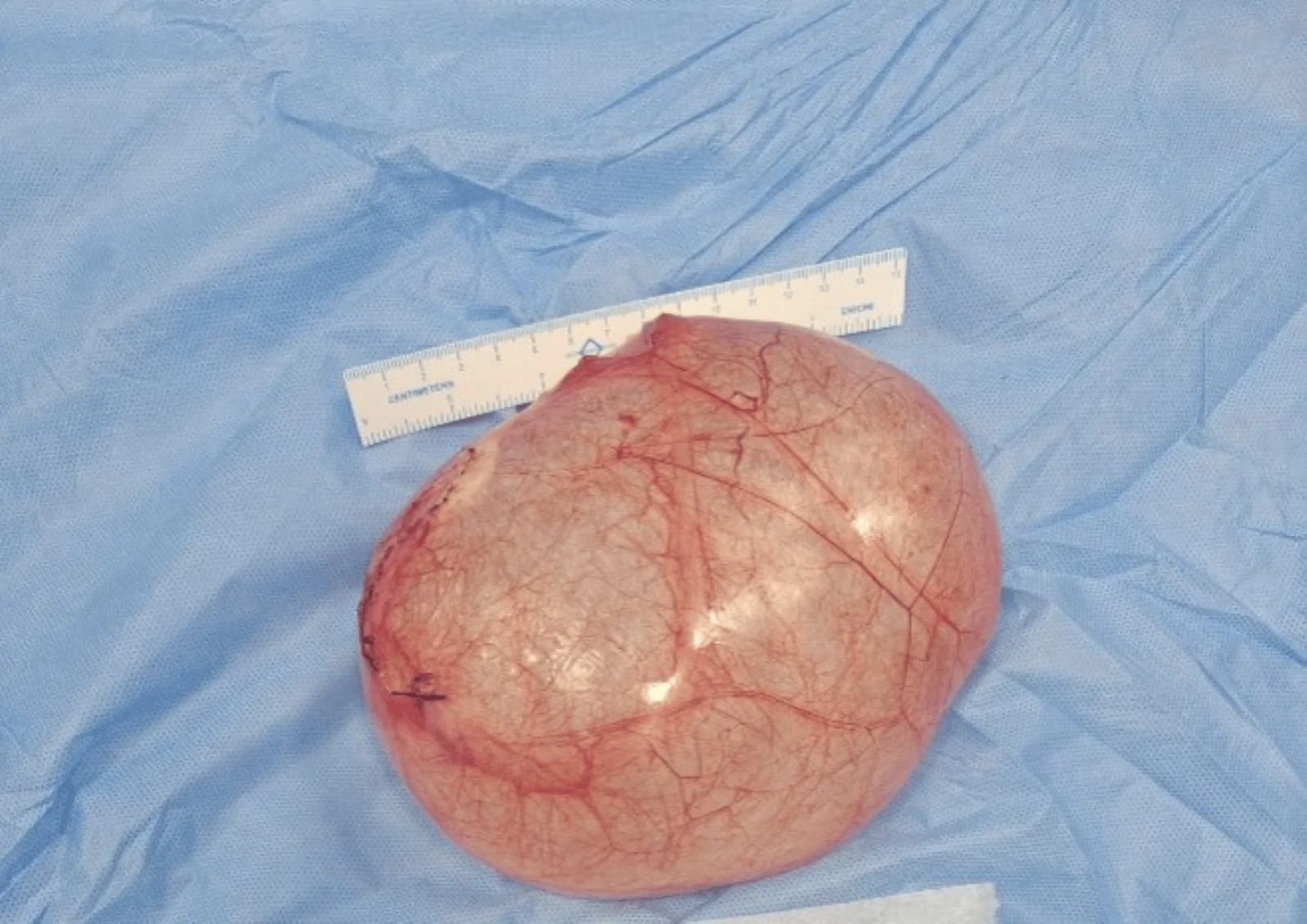
Pelvic cyst after excision

Histopathological examination

Gross examination revealed a cyst measuring 15 × 10.5 × 10 cm with a shiny congested external surface. On opening, the cyst contained clear serous fluid. A separate tube-like structure measuring approximately 1 cm in length was received along with the specimen.

Microscopic examination showed that the tube-like structure represented a normal fallopian tube. Sections from the cyst wall demonstrated fibrocollagenous tissue lined by cuboidal to columnar ciliated epithelium. No ovarian tissue or evidence of dysplasia or malignancy was identified.

Based on these findings, the final histopathological diagnosis was reported as a benign paratubal cyst with attached normal fallopian tube (Figure 6).

**Figure 6:**
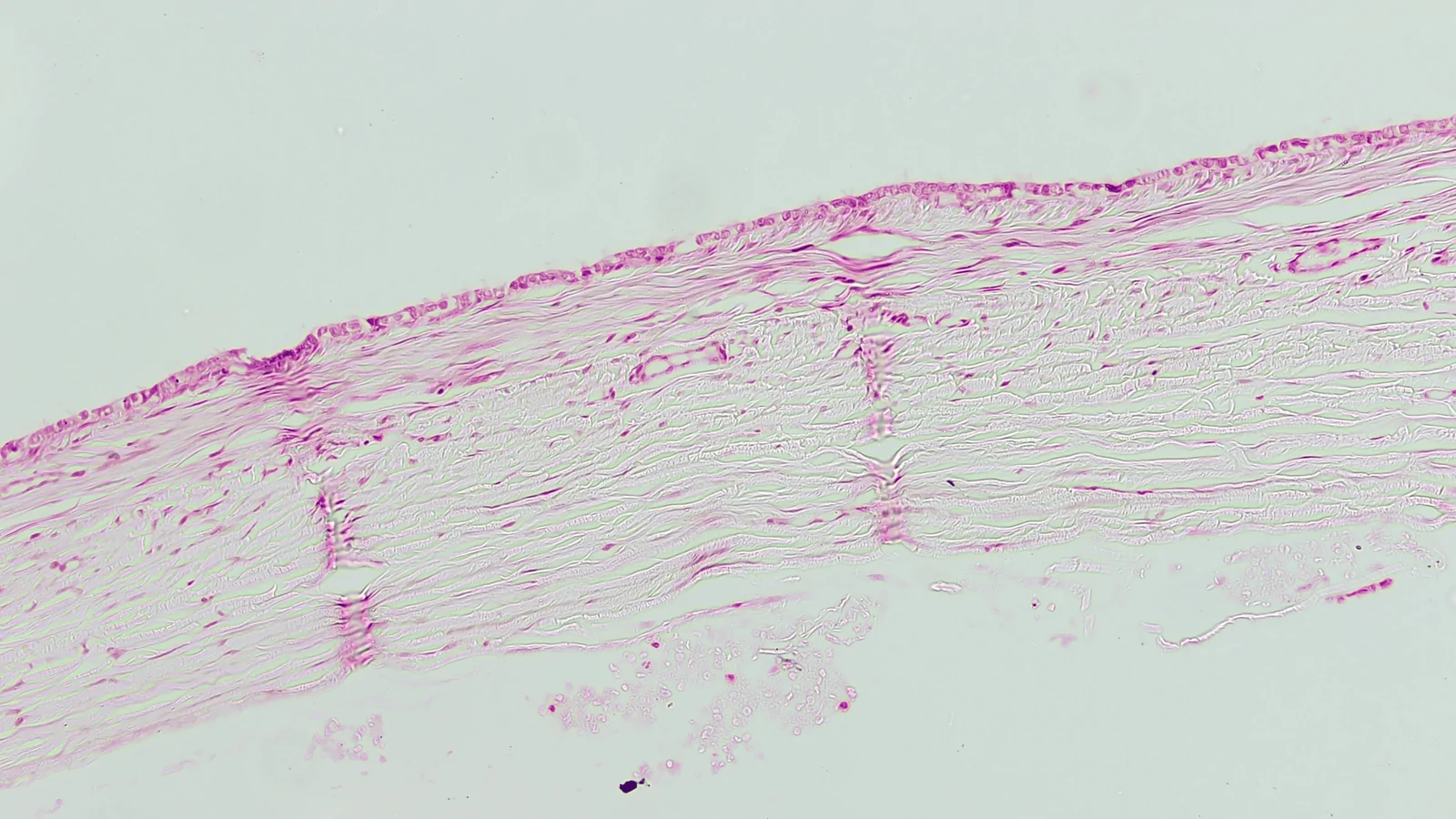
Histopathology image of the cyst

Correlation of operative and histopathological findings

The intraoperative impression suggested a lesion arising from the sigmoid mesentery because of its dense adherence to the sigmoid mesenteric border together with involvement of the left ovary and fimbrial end. However, histopathological examination established the lesion as a benign paratubal cyst. This discrepancy highlights the difficulty in determining the true origin of large pelvic cystic lesions solely on the basis of imaging or operative appearance when dense inflammatory adhesions distort normal anatomy.

Postoperative course

The postoperative period was uneventful. Oral intake was resumed after return of bowel function, pain was managed with standard analgesia, and the patient ambulated early. The abdominal wound healed well without evidence of surgical site infection. She was discharged in stable condition with advice for routine outpatient follow-up. At follow-up, she remained asymptomatic without evidence of recurrence.

## Discussion

Large pelvic cystic lesions continue to represent a diagnostic challenge because imaging modalities may fail to identify the precise anatomical origin when lesions become sufficiently large to distort adjacent pelvic anatomy. Tan et al. reported a giant intra-abdominal cyst arising from the sigmoid mesocolon in which the precise origin could not be determined preoperatively because of gross displacement of the surrounding viscera [11]. Similarly, Paramythiotis et al. emphasized that mesenteric cysts may be difficult to diagnose preoperatively because of the absence of pathognomonic clinical and imaging findings [15]. Although ultrasonography, MRI, and contrast-enhanced CT provide valuable information regarding lesion morphology and its relationship with surrounding structures, accurate determination of the organ of origin may remain difficult in giant pelvic cystic masses.

The present case differed from previously reported mesenteric cysts because dense adhesions between the cyst, sigmoid mesentery, left ovary, and fimbrial end strongly suggested a mesenteric origin during surgery. However, final histopathological examination established the diagnosis of a benign paratubal cyst with an attached normal fallopian tube. This discrepancy between the intraoperative impression and the definitive histopathological diagnosis emphasizes the limitations of relying solely on operative findings and highlights the importance of histopathological examination in establishing the final diagnosis.

Complete surgical excision is the preferred treatment for mesenteric cysts, particularly in symptomatic or large lesions, because it provides definitive treatment and reduces the risk of recurrence and complications [15]. When enucleation cannot be performed safely because of dense adhesions to surrounding tissues or organs, resection of adjacent structures may be required [15]. In the present patient, meticulous adhesiolysis permitted preservation of the sigmoid colon and left ovary while achieving complete excision with left fimbriectomy, resulting in an uneventful postoperative recovery.

This case further highlights the importance of multidisciplinary collaboration between gastrointestinal surgeons and gynecologists in the management of complex pelvic masses with uncertain origin. Such collaboration facilitates optimal operative planning, minimizes unnecessary organ resection, and ensures appropriate pathological correlation before establishing the final diagnosis.

A comparison of previously published literature on mesenteric cysts is added in Table 2. 

**Table 2:** Comparison with previous publications similar to the present case

Author	Diagnosis	Size	Surgical management	Similarity with present case
Tan et al. [11]	Benign extra-ovarian mucinous cystadenoma arising from the sigmoid mesocolon	21 × 25 × 26 cm	Laparotomy with en bloc complete excision	Giant intra-abdominal cyst with indeterminate preoperative origin and definitive diagnosis after surgery
Paramythiotis et al. [15]	Chylous (lymphatic) mesenteric cyst	7.08 × 6.05 cm	Open cystectomy with complete excision within healthy borders	Mesenteric cyst with difficulty in establishing the diagnosis preoperatively and successful complete surgical excision
Present case	Benign paratubal cyst with dense adhesions to the sigmoid mesentery	16 × 10 × 6 cm	Complete excision with left fimbriectomy	Intraoperative impression of mesenteric origin differed from the final histopathological diagnosis

## Conclusions

This case demonstrates the diagnostic complexity of giant pelvic cystic lesions. Although clinical examination, imaging, and intraoperative findings suggested a lesion arising from the sigmoid mesentery, final histopathological examination established the diagnosis of a benign paratubal cyst. Careful multidisciplinary planning, meticulous adhesiolysis, and complete surgical excision resulted in an excellent outcome. Histopathological correlation is essential whenever the intraoperative impression differs from the definitive tissue diagnosis.
